# In Vitro Activity of Cefiderocol, Eravacycline, and Imipenem–Relebactam Against Multidrug-Resistant *Acinetobacter baumannii* Clinical Isolates †

**DOI:** 10.3390/antibiotics15030246

**Published:** 2026-02-27

**Authors:** Yasemin Bölükbaşı, Betigül Öngen

**Affiliations:** 1 Medical Microbiology Department, İstanbul Faculty of Medicine, İstanbul University, 34093 İstanbul, Türkiye; ongenb@istanbul.edu.tr; 2 Medical Microbiology Department, Siirt Education and Research Hospital, 56100 Siirt, Türkiye

**Keywords:** *Acinetobacter baumannii*, cefiderocol, eravacycline, imipenem–relebactam, multidrug resistance, carbapenem resistance, broth microdilution, OXA-type carbapenemases

## Abstract

**Background/Objectives**: *Acinetobacter baumannii* is a leading cause of healthcare-associated infections and is frequently associated with multidrug resistance, severely limiting therapeutic options. The increasing prevalence of carbapenem-resistant *A. baumannii* (CRAB) has intensified interest in novel antimicrobial agents such as cefiderocol, eravacycline, and imipenem–relebactam. **Methods**: A total of 80 multidrug-resistant (MDR) *A. baumannii* isolates recovered from various clinical specimens between April 2019 and October 2023 were included. Antimicrobial susceptibility testing was performed using disk diffusion, gradient test, and broth microdilution methods in accordance with EUCAST and CLSI recommendations. The minimum inhibitory concentrations (MIC’s) for cefiderocol were evaluated with broth microdilution using iron-depleted cation-adjusted Mueller–Hinton broth as the reference method. The presence of carbapenem resistance–associated genes (*bla*OXA-23, *bla*OXA-24, *bla*OXA-51, *bla*OXA-58, *bla*IMP, and *tetA*) was investigated by polymerase chain reaction. **Results**: All isolates were resistant to imipenem and meropenem. Colistin resistance was detected in 7.5% of isolates. According to EUCAST criteria, cefiderocol susceptibility was observed in 77.5% of isolates by microdilution and in 81.25% by disk diffusion. Eravacycline demonstrated low MIC values, with MIC_50_ and MIC_90_ of 0.25 mg/L and 0.75 mg/L, respectively. In contrast, all isolates were resistant to imipenem–relebactam. The *bla*OXA-23 gene was detected in 82.5% and *bla*OXA-24 in 17.5% of isolates, while no *bla*OXA-58, *bla*IMP, or *tetA* genes were identified. No statistically significant association was found between cefiderocol resistance and OXA-type carbapenemase genes. **Conclusions**: Cefiderocol and eravacycline demonstrated promising in vitro activity against MDR *A. baumannii*, including colistin-resistant isolates, whereas imipenem–relebactam showed no activity. These findings support the potential role of cefiderocol and eravacycline as alternative treatment options for CRAB infections and highlight the multifactorial nature of cefiderocol resistance beyond OXA-type carbapenemase production.

## 1. Introduction

*Acinetobacter baumannii* is a clinically significant opportunistic pathogen responsible for a wide range of healthcare-associated infections, including pneumonia, bloodstream infections, urinary tract infections, and wound infections. Although less frequently, it may also cause community-acquired infections. The remarkable ability of *A. baumannii* to survive in hospital environments and to rapidly acquire antimicrobial resistance has led to its emergence as one of the most problematic nosocomial pathogens worldwide [1].

Outbreak-associated *A. baumannii* isolates are frequently resistant to carbapenems and often display reduced susceptibility to tigecycline. Multidrug-resistant (MDR) and extensively drug-resistant (XDR) *A. baumannii* strains typically remain susceptible only to a limited number of antimicrobial agents, most notably colistin. Consequently, treatment options for infections caused by carbapenem-resistant *A. baumannii* (CRAB) are severely restricted, resulting in increased morbidity and mortality, particularly among critically ill patients. In recognition of this global threat, the World Health Organization (WHO) classified CRAB as a “Priority 1: Critical pathogen” in its 2017 priority pathogens list, emphasizing the urgent need for the development of novel antimicrobial agents [2,3,4].

Surveillance data from the Central Asian and European Surveillance of Antimicrobial Resistance (CAESAR) and the European Antimicrobial Resistance Surveillance Network (EARS-Net) continue to demonstrate alarmingly high resistance rates among *Acinetobacter* species across Europe. Reports indicate that resistance to carbapenems, fluoroquinolones, and aminoglycosides remains widespread, with a substantial proportion of isolates exhibiting MDR [5,6]. The COVID-19 pandemic has further exacerbated this problem, particularly due to prolonged intensive care unit stays and increased antimicrobial exposure.

Colistin has long been considered a last-resort treatment for CRAB infections; however, its clinical utility is limited by nephrotoxicity, suboptimal tissue penetration, and the emergence of colistin-resistant isolates. Reports from multiple regions worldwide, including Asia and Europe, have documented increasing rates of colistin resistance, raising serious concerns regarding the sustainability of current treatment strategies [7,8,9,10,11,12,13]. In Türkiye, colistin resistance among *A. baumannii* isolates has been reported at variable rates, generally ranging between 1% and 7%, highlighting the need for alternative therapeutic options [14,15,16,17,18].

In response to the escalating resistance crisis, several novel antimicrobial agents have been developed and approved in recent years. Cefiderocol, a siderophore cephalosporin with a unique iron-transport–mediated mechanism of entry, has demonstrated potent in vitro activity against a broad range of Gram-negative pathogens, including CRAB. Eravacycline, a synthetic fluorocycline antibiotic, has shown enhanced in vitro activity compared with tigecycline against MDR and XDR *A. baumannii* [19,20]. In contrast, the clinical relevance of imipenem–relebactam against *A. baumannii* remains controversial due to intrinsic resistance mechanisms and limited inhibitor activity against OXA-type carbapenemases.

Accurate antimicrobial susceptibility testing for these agents, particularly cefiderocol, presents methodological challenges. Cefiderocol testing requires iron-depleted conditions to reliably reflect its siderophore-mediated activity, and discrepancies between broth microdilution and disk diffusion methods have been reported. Furthermore, the contribution of specific resistance mechanisms, including OXA-type carbapenemases and other chromosomal factors, to cefiderocol resistance remains incompletely understood.

This study aimed to evaluate the in vitro activity of cefiderocol, eravacycline, and imipenem–relebactam against MDR *A. baumannii* clinical isolates. We also explored the association between antimicrobial susceptibility profiles and the presence of carbapenem resistance–associated genes, with a particular emphasis on OXA-type carbapenemases. The novelty of this study lies in the integrated evaluation of cefiderocol susceptibility testing methodologies, the regional epidemiology of CRAB in Türkiye, and the underlying molecular resistance mechanisms, as well as the parallel assessment of three novel antimicrobial agents within the same *A. baumannii* isolate collection.

## 2. Results

### 2.1. Characteristics of Patients from Whom the Strains Were Isolated

The isolates were recovered from patients with a mean age of 46.6 years (median age: 59 years); 63.8% of patients were male and 36.2% were female. Most isolates were obtained from hospitalized patients (88.75%), while 11.25% originated from outpatient settings. Among hospitalized patients, 46.25% were admitted to intensive care units.

Respiratory specimens constituted the majority of clinical samples, with sputum (37.5%) and tracheal aspirates (11.25%) being the most frequent sources, followed by blood (16.25%), tissue samples (12.5%), and urine (10%).

### 2.2. Identification and Antimicrobial Susceptibility Profiles

All isolates were identified as *A. baumannii* using the VITEK-2 system. Antimicrobial susceptibility testing revealed that all isolates were resistant to imipenem and meropenem. High resistance rates were also observed for ciprofloxacin (98.75%), piperacillin–tazobactam (98.75%), trimethoprim–sulfamethoxazole (90%), and amikacin (88.75%). Colistin resistance was detected in 7.5% (6/80; 95% CI: 3.5–15.2%) of isolates.

Tigecycline MIC values were evaluated based on EUCAST pharmacokinetic/pharmacodynamic breakpoints and for descriptive comparison only. MIC values were distributed as follows: <0.5 mg/L in 13 isolates, 1 mg/L in 19 isolates, 2 mg/L in 19 isolates, 4 mg/L in 27 isolates, and >4 mg/L in 2 isolates.

### 2.3. Cefiderocol Susceptibility Testing

#### 2.3.1. Disk Diffusion and Gradient Test Results

Using EUCAST v14.0 criteria, disk diffusion testing with a 30 µg cefiderocol disk identified 65 (81.25%) isolates as susceptible based on zone diameters ≥17 mm. Fifteen isolates (18.75%) exhibited zone diameters <17 mm and were classified as resistant. The MIC values among these resistant isolates ranged from 0.5 mg/L to >256 mg/L, and MIC values were as follows: >256 mg/L (*n* = 5), 128 mg/L (*n* = 4), 16 mg/L (*n* = 1), 8 mg/L (*n* = 2), 2 mg/L (*n* = 1), 0.5 mg/L (*n* = 2).

According to CLSI M100 (33rd edition) [21], 12 isolates (15%) exhibited zone diameters ≤14 mm. MIC values were as follows; >256 mg/L (*n* = 5), 128 mg/L (*n* = 4), 16 mg/L (*n* = 1), 8 mg/L (*n* = 1), and 0.5 mg/L (*n* = 1).

Microcolonies were observed within the inhibition zone in 12 isolates. Among these, three isolates demonstrated zone diameters ≥17 mm but exhibited MIC values of 1 mg/L, 2 mg/L, and 128 mg/L, respectively. The remaining nine isolates had zone diameters <17 mm; MIC values were determined as >256 mg/L (*n* = 3), 128 mg/L (*n* = 2), 16 mg/L (*n* = 1), 8 mg/L (*n* = 1), 2 mg/L (*n* = 1), and 0.5 mg/L (*n* = 1).

Gradient test validation experiments demonstrated that this method was not reliable for determining cefiderocol susceptibility in *Acinetobacter* spp.; therefore, gradient testing was excluded from further analyses.

#### 2.3.2. Broth Microdilution Results

Using the reference broth microdilution method with iron-depleted cation-adjusted Mueller–Hinton broth, 62 isolates (77.5%; 95% CI: 67.3–85.2%) were categorized as susceptible to cefiderocol according to EUCAST criteria, while 18 isolates (22.5%) were resistant (MIC >2 mg/L). MIC values among resistant isolates were distributed as follows: >256 mg/L (*n* = 7), 128 mg/L (*n* = 6), 16 mg/L (*n* = 1), 8 mg/L (*n* = 2), and 4 mg/L (*n* = 2).

Based on CLSI criteria, 64 isolates (80%) were classified as susceptible, 2 isolates (2.5%) as susceptible with increased exposure, and 14 isolates (17.5%) as resistant.

**Figure 1:**
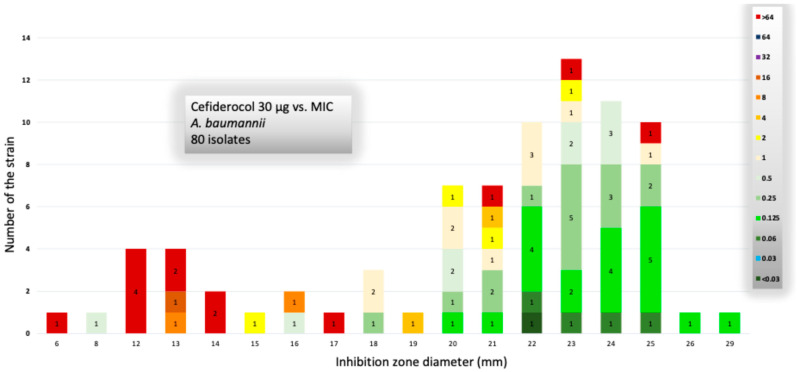
Distribution of cefiderocol resistance pattern.

The MIC_50_ and MIC_90_ values for cefiderocol were 0.25 mg/L and 128 mg/L, respectively, with a MIC range of 0.06–256 mg/L. The comparative distribution of cefiderocol zone diameters and MIC values according to EUCAST criteria is presented in Figure 1.

Using broth microdilution as the reference method, the categorical agreement between disk diffusion and broth microdilution for cefiderocol was 96.25%. No major errors were observed. Three isolates categorized as susceptible by disk diffusion were resistant by broth microdilution, resulting in a very major error rate of 16.7% (Table 1).

### 2.4. Eravacycline Susceptibility Results

Eravacycline susceptibility results were reported as MIC values without categorical interpretation, in accordance with EUCAST recommendations. Disk diffusion testing yielded zone diameters ranging from 13 to 27 mm. Gradient testing demonstrated MIC values between 0.016 mg/L and 0.75 mg/L “see Supplementary Materials”.

The MIC_50_ and MIC_90_ values for eravacycline were 0.25 mg/L and 0.75 mg/L, respectively.

### 2.5. Comparison of Eravacycline and Tigecycline MIC Values

Among isolates with a tigecycline MIC of 2 mg/L (*n* = 19), eravacycline MIC values ranged from 0.125 mg/L to 0.50 mg/L. In isolates with a tigecycline MIC of 4 mg/L (*n* = 27), eravacycline MIC values ranged from 0.19 mg/L to 0.75 mg/L. “see Supplementary Materials”.

### 2.6. Imipenem–Relebactam Susceptibility Results

Disk diffusion testing demonstrated growth up to the disk edge (6 mm) in 64 isolates, all of which exhibited MIC values >32 mg/L. The remaining isolates showed limited inhibition zones, with corresponding MIC values ranging from 12 to 32 mg/L. No isolate demonstrated susceptibility to imipenem–relebactam.

### 2.7. Colistin Susceptibility Results

Colistin resistance was detected in six isolates (7.5%). MIC values ranged from <0.25 mg/L to >16 mg/L. One isolate yielded inconsistent MIC results despite repeated testing and was recorded as <0.25 mg/L.

### 2.8. Resistance Genes Profile

PCR analysis confirmed the presence of the intrinsic *bla*OXA-51 gene in all isolates. The *bla*OXA-23 gene was detected in 66 isolates (82.5%), while *bla*OXA-24 was identified in 14 isolates (17.5%). No isolate carried *bla*OXA-58, *bla*IMP, or *tetA* genes.

No statistically significant association was observed between cefiderocol resistance and the presence of *bla*OXA-23 and *bla*OXA-24 genes (*p* = 0.679, and *p* = 0.311 respectively).

## 3. Discussion

The rapid global dissemination of MDR *A. baumannii* continues to pose a major therapeutic challenge, particularly in healthcare-associated infections involving critically ill patients [1]. In the present study, we evaluated the in vitro activity of cefiderocol, eravacycline, and imipenem–relebactam against CRAB clinical isolates and investigated the relationship between antimicrobial susceptibility patterns and carbapenem resistance–associated genes.

Consistent with surveillance data from Europe and other regions, all isolates included in this study were resistant to carbapenems, confirming the high prevalence of CRAB in tertiary-care hospital settings [5,6]. Resistance rates to commonly used antimicrobial agents such as fluoroquinolones, aminoglycosides, and trimethoprim–sulfamethoxazole were also remarkably high, leaving few effective therapeutic options. Colistin resistance was detected in 7.5% of isolates, a rate comparable to previously reported data from Türkiye and neighboring countries, underscoring the ongoing but still limited emergence of colistin resistance [16,17,18].

Cefiderocol demonstrated notable in vitro activity against the majority of isolates. Using the reference broth microdilution method under iron-depleted conditions, 77.5% of isolates were classified as susceptible according to EUCAST criteria, while disk diffusion testing yielded slightly higher susceptibility rates. This discrepancy aligns with previous reports indicating that disk diffusion may overestimate cefiderocol susceptibility, particularly in isolates with elevated MIC values [22,23]. The methodological challenges associated with cefiderocol susceptibility testing, including endpoint interpretation and the requirement for iron-depleted media, remain important considerations for routine laboratory implementation [24,25].

Microcolonies observed during disk diffusion testing further complicate the interpretation of cefiderocol susceptibility. In our study, several isolates exhibited intrazonal growth. The tests were performed twice, according to EUCAST recommendations, and the same results were obtained. The isolates exhibiting intrazonal growth displayed discordant MIC values, reinforcing concerns regarding categorical agreement between disk diffusion and broth microdilution methods. The presence of microcolonies within inhibition zones, even in isolates with low MIC values, may reflect heteroresistance or subpopulations with altered iron uptake mechanisms, rather than true categorical susceptibility. However, methodological factors inherent to diffusion-based testing of siderophore antibiotics may also contribute to this phenomenon [26,27].

In line with current EUCAST recommendations, isolates exhibiting intrazonal growth were interpreted as resistant in our study. Overall, these findings support the use of broth microdilution performed under iron-depleted conditions as the reference method for cefiderocol susceptibility testing in *A. baumannii*, particularly when disk diffusion results are borderline or show microcolonies.

From a laboratory perspective, disk diffusion results, especially those showing intrazonal growth or borderline zone diameters, should be interpreted with caution and ideally confirmed by broth microdilution to avoid misleading susceptibility categorization.

Eravacycline demonstrated consistently low MIC values across all isolates, including those resistant to colistin and carbapenems. The observed MIC_50_ and MIC_90_ values were lower than those reported for tigecycline, supporting previous evidence that eravacycline exhibits enhanced in vitro activity against MDR and XDR *A. baumannii* [20,28]. Although eravacycline exhibited low MIC values in this study, the lack of established clinical breakpoints for *A. baumannii* limits clinical interpretation. Therefore, these findings should be considered descriptive in vitro data observations rather than indicators of clinical efficacy. While eravacycline demonstrates favorable pharmacokinetic properties, its clinical role in CRAB infections requires further clinical and PK/PD-supported investigation. The MIC distribution observed in this study suggests potential activity under laboratory conditions, particularly in settings with limited treatment options [29].

In contrast, imipenem–relebactam showed no in vitro activity against any of the isolates tested. This finding is in line with existing data indicating that relebactam does not inhibit OXA-type carbapenemases, which are the predominant resistance mechanism in *A. baumannii*. The absence of metallo-β-lactamase genes in our isolates further supports the conclusion that intrinsic chromosomal resistance mechanisms likely account for the observed lack of activity [30].

The molecular analysis revealed a high prevalence of *bla*OXA-23, consistent with global and regional reports identifying this enzyme as the dominant carbapenemase among CRAB isolates [31,32]. Although *bla*OXA-24 was detected less frequently, its presence was associated with higher cefiderocol MIC_90_ values, suggesting potential differences in resistance phenotypes that warrant further investigation. However, the limited number of isolates carrying *bla*OXA-24 precludes definitive conclusions. The association between cefiderocol resistance and carbapenemase genes was also evaluated in *A. baumannii* isolates. No statistically significant relationship was detected between cefiderocol resistance and the presence of *bla*OXA-23 or *bla*OXA-24. This finding suggests that cefiderocol resistance may not be explained solely by the presence of specific OXA-type β-lactamases. Beyond OXA-type carbapenemases, several biologically plausible mechanisms may contribute to reduced cefiderocol susceptibility in *A. baumannii*. Alterations in siderophore-mediated iron transport systems, mutations affecting TonB-dependent receptors, overexpression of efflux pumps, and changes in outer membrane permeability have all been proposed as potential contributors to decreased activity of cefiderocol. In addition, adaptive responses related to iron homeostasis and heteroresistant subpopulations may further complicate susceptibility interpretation. These multifactorial mechanisms highlight that cefiderocol resistance is likely not driven by a single determinant but rather by complex interactions between bacterial physiology and testing methodology. Similar observations have been reported in previous studies, highlighting the multifactorial nature of cefiderocol resistance in *A. baumannii* [33,34]. Therefore, we believe that comprehensive molecular approaches, such as whole-genome sequencing and detailed analysis of iron acquisition pathways, are necessary to elucidate the mechanisms underlying cefiderocol resistance more fully. Another factor contributing to this result is the limited number of cefiderocol-resistant isolates, which may have reduced the statistical power to detect an association with OXA-type carbapenemases.

This study has several limitations. First, the findings are based solely on in vitro antimicrobial susceptibility data, and clinical outcomes such as treatment response, microbiological eradication, or patient survival were not assessed. Therefore, no direct correlation between in vitro susceptibility results and clinical efficacy can be established. Second, it represents data from a single tertiary-care center with a limited number of isolates which may restrict generalizability of the results. In particular, the limited number of cefiderocol-resistant isolates may have reduced the statistical power to detect an association with OXA-type carbapenemases. Third, whole-genome sequencing and clonal analysis were not performed, preventing a more comprehensive assessment of resistance mechanisms and molecular epidemiology.

Future studies should focus on prospective, multicenter investigations that integrate antimicrobial susceptibility data with clinical outcomes, pharmacokinetic/pharmacodynamic parameters, and molecular resistance mechanisms. Such studies would provide a more comprehensive understanding of the clinical relevance of cefiderocol and eravacycline in the treatment of CRAB infections.

Despite these limitations, our findings provide valuable insight into the in vitro activity of novel antimicrobial agents against MDR *A. baumannii*. Cefiderocol and eravacycline demonstrated promising activity, whereas imipenem–relebactam was ineffective. These results support the continued evaluation of cefiderocol and eravacycline as potential treatment options for CRAB infections and emphasize the need for further multicenter and clinical studies to better define their role in therapy.

## 4. Materials and Methods

### 4.1. Study Design

Based on the sample size calculation, a total of 80 MDR *Acinetobacter* spp. isolates were included in this study. The isolates were recovered from various clinical specimens (blood, tracheal aspirate, sputum, sterile body fluids, abscesses, tissue, urine, etc.) collected between April 2019 and October 2023 from pediatric and adult patients followed at medical and surgical departments or outpatient clinics at Istanbul Faculty of Medicine Hospital. When multiple isolates were obtained from the same patient, only the first isolate was included in the study.

All isolates were stored at −80 °C in broth containing 20% glycerol. Prior to testing, frozen isolates were subcultured twice on tryptic soy agar (TSA) and incubated at 37 °C for 18–24 h to obtain pure cultures.

### 4.2. Bacterial Identification and Routine Antimicrobial Susceptibility Testing

Species-level identification and routine antimicrobial susceptibility testing were performed using the Vitek-2 automated system (bioMérieux, Marcy-l’Étoile, France). Due to the limited reliability of automated systems for colistin susceptibility testing, colistin MICs were determined using the reference broth microdilution method [35].

Disk diffusion and gradient test methods were applied for eravacycline (20 µg) and imipenem–relebactam [35 (10/25) µg], whereas cefiderocol (30 µg) susceptibility was evaluated using disk diffusion and broth microdilution methods.

### 4.3. Disk Diffusion and Gradient Test Methods

Bacterial suspensions equivalent to 0.5 McFarland standard were prepared from fresh cultures and inoculated onto Mueller–Hinton agar (MHA) plates. Eravacycline, imipenem–relebactam, and cefiderocol disks (Liofilchem, Roseto degli Abruzzi, Italy) were placed on the agar surface.

Gradient tests for eravacycline and imipenem–relebactam were performed using gradient strips (Liofilchem, Roseto degli Abruzzi, Italy) applied in reverse orientation on a second MHA plate. Plates were incubated at 35 ± 1 °C for 18 ± 2 h. After incubation, inhibition zone diameters and MIC values at the point where the inhibition ellipse intersected the strip were recorded.

Results were interpreted according to EUCAST and CLSI criteria and categorized as susceptible or resistant [21,36].

### 4.4. Broth Microdilution Method for Cefiderocol

Cefiderocol susceptibility testing was performed using the broth microdilution method in iron-depleted cation-adjusted Mueller–Hinton broth (ID-CAMHB). In the process of preparing ID-CAMHB, 100 g of chelating resin (Bio-rad, Hercules, CA, USA) was added to 1 L of CAMHB and mixed with a magnetic stir bar at room temperature for approximately 2 h. Afterward, it was filtered using a 0.2 μm filter. Finally cations (Ca^++^, Mg^++^, Zn^++^) were added to achieve the final concentration range. The medium was prepared to achieve a free iron concentration of ≤0.03 µg/mL [37].

Cefiderocol powder (Sigma-Aldrich, St. Louis, MO, USA) was diluted in twofold serial concentrations ranging from 64 mg/L to 0.03 mg/L in 96-well microplates. Plates were incubated at 35 ± 1 °C for 18 ± 2 h. The MIC was defined as the lowest concentration with no visible growth. Susceptibility categories were determined according to EUCAST breakpoint table version 14.0 and CLSI M100 (33rd edition).

### 4.5. Broth Microdilution Method for Colistin

Colistin MICs were determined using a commercial MIC COL kit (Diagnostics Inc., Galanta, Slovakia) according to the manufacturer’s instructions.

### 4.6. Quality Control

*Escherichia coli* ATCC 25922 and *Pseudomonas aeruginosa* ATCC 27853 were used as quality control strains in all susceptibility testing procedures.

### 4.7. Molecular Analysis

The resistance genes included in the PCR analysis were selected based on their reported epidemiological relevance and biological plausibility in *A. baumannii*. OXA-type carbapenemases (*bla*OXA-23, *bla*OXA-24, and *bla*OXA-51) were targeted due to their predominant role in carbapenem resistance in Türkiye and globally. Additional genes, including *bla*OXA-58 and *bla*IMP, were investigated to explore alternative resistance mechanisms, while tetA was included to evaluate potential tetracycline-related resistance determinants relevant to fluorocycline activity. The primers used in the study are shown in Table 2.

To detect six antimicrobial resistance genes in *A. baumannii* isolates, three different (one multiplex and two monoplex) PCR amplification protocols were applied. The *bla*OXA-23, *bla*OXA-51, *bla*OXA-58, and *tetA* genes were investigated using multiplex PCR-1, while the *bla*IMP gene was analyzed using PCR-2. The PCR-1 amplification program was as follows: at 94 °C for 5 min, 35 cycles at 94 °C for 25 s, at 52 °C for 40 s, at 72 °C for 50 s, and lastly at 72 °C for 6 min. PCR-2 amplification program was as follows: at 94 °C for 5 min, 35 cycles at 94 °C for 25 s, at 50.2 °C for 40 s, at 72 °C for 50 s, and lastly at 72 °C for 6 min. Due to insufficient amplification of the *bla*OXA-24 gene under multiplex PCR conditions, an optimized monoplex PCR protocol was applied separately for this gene. The amplification program was as follows: at 94 °C for 5 min, 35 cycles at 94 °C for 25 s, at 56.3 °C for 40 s, at 72 °C for 50 s, and lastly at 72 °C for 6 min.

Genomic DNA was extracted using the boiling method. PCR cycling conditions were established based on the protocols described by Woodford et al. [38]. PCR products were analyzed by gel electrophoresis at 120 V for 25 min and visualized under UV transillumination.

### 4.8. Sample Size and Statistical Analysis

Based on the literature, a sample size of *n* = 77 was calculated using the G*Power software (version 3.1.9.7), assuming an effect size of 0.38, a power of 95%, and a margin of error of 0.5. As a single-group analysis was planned, the One-Sample Case test approach was used, and a total of 80 isolates were included in the study.

Statistical analyses were performed using IBM SPSS Statistics (version 29). Categorical variables were expressed as numbers and percentages. The association between resistance gene presence and cefiderocol resistance was evaluated using Fisher’s exact test. Comparisons of cefiderocol and colistin susceptibility rates within the same isolates were performed using McNemar’s test. A *p* value < 0.05 was considered statistically significant.

Fisher’s exact test was used for gene–phenotype association analyses due to small expected cell counts in contingency tables. McNemar’s test was applied to compare paired antimicrobial susceptibility outcomes within the same isolates.

## 5. Conclusions

In this study, cefiderocol and eravacycline demonstrated promising in vitro activity against MDR A. baumannii clinical isolates, including colistin-resistant strains, whereas imipenem–relebactam showed no activity. Cefiderocol resistance was not significantly associated with OXA-type carbapenemase genes, suggesting that resistance is likely multifactorial and not solely dependent on β-lactamase production. These findings highlight the potential role of cefiderocol and eravacycline as alternative treatment options for CRAB infections and underscore the need for further multicenter and clinical studies to better define their therapeutic utility.

## Figures and Tables

**Table 1:** The categorical agreement and error rates between disk diffusion and broth microdilution for cefiderocol.

Parameter	Value (%)
Categorical agreement	96.25
Major error (ME)	0
Very major error (VME)	16.7

**Table 2:** Primers used for PCR assays.

Target Genes	Primers (5′-3′)
*bla*OXA-23	F: GAT CGG ATT GGA GAA CCA GA
	R: ATT TCT GAC CGC ATT TCC AT
*bla*OXA-24	F: GGT TAG TTG GCC CCC TTA AA
	R: AGT TGA GCG AAA AGG GGA TT
*bla*OXA-51	F: TAA TGC TTT GAT CGG CCT TG
	R: TGG ATT GCA CTT CAT CTT GG
*bla*OXA-58	F: AAG TAT TGG GGC TTG TGC TG
	R: CCC CTC TGC GCT CTA CAT AC
*bla*IMP	F: CAT GGT TTG GTG GTT CTT GT
	R: ATA ATT TGG CGG ACT TTG GC
*tet-A*	F: GCT ACA TCC TGC TTG CCT TC
	R: CAT AGA TCG CCG TGA AGA GG

## Data Availability

The data presented in this study are available from the corresponding author upon reasonable request.

## Supplementary Materials

The following supporting information can be downloaded at: https://www.mdpi.com/article/10.3390/antibiotics15030246/s1, Table S1: Distribution of eravacyclin (ERV) zone diameter and MIC values.; Table S2: Comparison of eravacycline (ERV) MIC—zone diameter distribution with tigecycline (TGC) MIC results.
