# Supplementary material for: In Vitro Activity of Cefiderocol, Eravacycline, and Imipenem–Relebactam Against Multidrug-Resistant Acinetobacter baumannii Clinical Isolates
Source: Antibiotics (Basel). 2026 Feb 27;15(3):246. doi: 10.3390/antibiotics15030246 (PMC13024395; doi:10.3390/antibiotics15030246)
Supplement: Supplementary file 1 [file antibiotics-15-00246-s001.zip › Supplementary materials Table S1.pdf]

Table S1. Distribution of eravacyclin (ERV) zone diameter and MIC values.

**ERV zone diameters (mm)**	**ERV MIC (mg/L)**										
	0,016	0,023	0,032	0,064	0,094	0,125	0,19	0,25	0,38	0,5	0,75
13									1		
14										1	
15								1		1	1
16							1	1	1	10	3
17						1	2	4	6	4	
18							7	9	2	2	
19						3	2	3	1		
20					1		3				
21					1						
22			1		1						
23		1		1	1		1				
24					1						
27	1										

ERV: Eravacyline
