# Supplementary material for: In Vitro Activity of Cefiderocol, Eravacycline, and Imipenem–Relebactam Against Multidrug-Resistant Acinetobacter baumannii Clinical Isolates
Source: Antibiotics (Basel). 2026 Feb 27;15(3):246. doi: 10.3390/antibiotics15030246 (PMC13024395; doi:10.3390/antibiotics15030246)
Supplement: Supplementary file 1 [file antibiotics-15-00246-s001.zip › Supplementary materials Table S2.pdf]

Table S2. Comparison of eravacycline (ERV) MIC - zone diameter distribution with tigecycline (TGC) MIC results.

		ERV zone diameters (mm/n)													Total (n)
**TGC MIC**	**ERV MIC**	**13**	**14**	**15**	**16**	**17**	**18**	**19**	**20**	**21**	**22**	**23**	**24**	**27**	
**<0.5**															**13**
	0.023											1			1
	0.064											1			1
	0.094											1			1
	0.125							2							2
	0.19						1	1				1			3
	0.25						3	1							4
	0.94												1		1
**1**															**19**
	0.016													1	1
	0.032										1				1
	0.094								1	1					2
	0.19				1		3		3						7
	0.25					2	1	1							4
	0.38					3									3
	0.50				1										1
**2**															**19**
	0.125					1		1							2
	0.19					1	1								2
	0.25			1	1	2	3	1							8
	0.38					2									2
	0.50				3	2									5
**4**															**27**
	0.19					1	2	1							4
	0.25						2								2
	0.38	1			1	1	2	1							6
	0.50		1	1	6	2	2								12
	0.75			1	2										3
**>4**															**2**
	0.094										1				1
	0.75				1										1
**Total (n)**		**1**	**1**	**3**	**16**	**17**	**20**	**9**	**4**	**1**	**2**	**4**	**1**	**1**	**80**
